# Increasing Cervical Cancer Awareness and Screening in Jamaica: Effectiveness of a Theory-Based Educational Intervention

**DOI:** 10.3390/ijerph13010053

**Published:** 2015-12-22

**Authors:** Evelyn Coronado Interis, Chidinma P. Anakwenze, Maug Aung, Pauline E. Jolly

**Affiliations:** 1 School of Public Health, University of Alabama at Birmingham, Birmingham, AL 35294, USA; jollyp@uab.edu; 2 School of Medicine, University of Alabama at Birmingham, Birmingham, AL 35294, USA; chidi89@uab.edu; 3 Epidemiology and Research Unit, Western Regional Health Authority, Montego Bay, Jamaica; maung.aung@wrha.gov.jm

**Keywords:** cervical cancer, screening, health promotion, intervention, risk, prevention, disease control, health behavior

## Abstract

Despite declines in cervical cancer mortality in developed countries, cervical cancer incidence and mortality rates remain high in Jamaica due to low levels of screening. Effective interventions are needed to decrease barriers to preventive behaviors and increase adoption of behaviors and services to improve prospects of survival. We enrolled 225 women attending health facilities in an intervention consisting of a pre-test, educational presentation and post-test. The questionnaires assessed attitudes, knowledge, risk factors, and symptoms of cervical cancer among women. Changes in knowledge and intention to screen were assessed using paired *t*-tests and tests for correlated proportions. Participants were followed approximately six months post-intervention to determine cervical cancer screening rates. We found statistically significant increases from pre-test to post-test in the percentage of questions correctly answered and in participants’ intention to screen for cervical cancer. The greatest improvements were observed in responses to questions on knowledge, symptoms and prevention, with some items increasing up to 62% from pre-test to post-test. Of the 123 women reached for follow-up, 50 (40.7%) screened for cervical cancer. This theory-based education intervention significantly increased knowledge of and intention to screen for cervical cancer, and may be replicated in similar settings to promote awareness and increase screening rates.

## 1. Introduction

Cervical cancer is the second most common cancer in women in the developing world and the largest cancer killer among women in most developing countries, with 88% of deaths occurring in low-resource settings [1]. Jamaica has one of the highest cervical cancer incidence rates in the Americas, with an age-standardized incidence rate of 46 cases per 100,000 women, compared to 20.8 cases per 100,000 women in the Caribbean and 5.7 cases per 100,000 women in North America [2]. Furthermore, Jamaican women are more than four times as likely to die from cervical cancer as women living in the United States [3].

Papanicolaou cytology screening (Pap test) has dramatically decreased cervical cancer incidence and mortality in most developed countries, reducing mortality by approximately 99% among women who are screened regularly [4,5]. The high cervical cancer mortality in developing countries is largely attributed to ineffective screening programs, limited access to cervical cancer screening, and low levels of follow-up treatment after abnormal test results, an essential step in preventing cancer death [3,6].

Cervical cancer screening coverage in Jamaica remains low. A 2008 study found 66% of Jamaican women had undergone a Pap test at some point in the past [7]. However, only 6.3% of women aged 25 to 54 in Jamaica’s western parish of Trelawny had undergone a Pap test in the previous year [8]. This highlights missed opportunities for prevention among the large percentage of women who fail to follow Jamaica Cancer Society recommendations of undergoing screening once per year. The elevated cervical cancer burden and low participation in prevention services among Jamaican women indicate a need for interventions that increase awareness and improve cancer screening rates [9].

Obstacles to seeking cervical cancer screening in Jamaica include inadequate cervical cancer knowledge; lack of knowledge of where to seek screening; fear of pain or embarrassment from the procedure; limited access to quality health services, especially in rural and low-resource communities; lack of healthcare provider recommendations; and staff shortages in government laboratories that cause delays in receiving test results, lengthen the time between diagnosis and treatment, and lead to patients being lost to follow-up [3,8,10,11,12].

Programs emphasizing the benefits of screening have been shown to positively influence women’s cancer screening behavior and are needed to increase public awareness [8,12]. The World Health Organization recommends the implementation of cost-effective and affordable interventions to address cervical cancer, stressing early detection as the cornerstone strategy for controlling cervical cancer and improving health outcomes and survival [13]. The objectives of this study were to: (1) evaluate the effectiveness of an educational intervention based on the Transtheoretical and Health Belief Models of behavior change in increasing cervical cancer knowledge and intention to screen; and (2) assess cervical cancer screening rates post-intervention among study participants in western Jamaica.

## 2. Experimental Section

The study was conducted in the four parishes served by the Western Regional Health Authority (WRHA), with the aim of obtaining a sample reflective of the population of each parish. The parish of St James accounts for 39% of the western region population with 182,600 residents, followed by Westmoreland with 31% (141,800), Trelawny with 16% (72,500), and Hanover with 14% (67,200) [14]. WRHA is administered by the Jamaica Ministry of Health and provides free health services, including Pap tests, via a network of four hospitals and 84 health centers located throughout the region.

### 2.1. Study Design

We conducted a cross-sectional study with a pre-test/post-test design in the four WRHA hospitals and in non-randomized WRHA health centers (*n* = 5) and community sites (churches and health fairs, *n* = 5) in western Jamaica. We enrolled 225 women in the intervention from June to August 2013. The study population included women 18 years and older residing in the western region who had never had a Pap test or had not had one in five years or more. Five years was defined as the cutoff signifying women who no longer seek Pap tests on a regular basis.

The study population consisted of a convenience sample of women attending health facilities and community events. Women at hospital and health center sites were approached in waiting rooms by a member of the research team and were asked if they would like to participate in a study promoting cervical cancer awareness and screening. Women at community sites were approached in a similar fashion. Women who met the inclusion criteria and expressed interest in participating were taken through the informed consent process. Written informed consent was obtained from each participant prior to commencement of the intervention.

The intervention was based on constructs from two health behavior change theories, the Transtheoretical Model (TTM) and the Health Belief Model (HBM), which have been used successfully in promoting positive cancer screening behavior in similar settings [15,16,17]. The TTM assesses an individual’s readiness to enact behavior change and provides strategies to help individuals move through the Stages of Change—pre-contemplation, contemplation, preparation, action, and maintenance—under the premise that individuals respond to consciousness-raising to adopt protective health behaviors [18]. The HBM helps identify barriers and facilitators for adopting behavior change [19], and was used in the intervention to emphasize benefits of screening while minimizing barriers.

The intervention instruments consisted of an educational presentation and a pre-test/post-test questionnaire, developed by the research team, and informed by findings from previous cancer studies conducted in Jamaica [3,8,15] and educational information and guidelines promoted by the Jamaica Cancer Society. The instruments were developed for an audience with a reading comprehension level below the 8th grade. Participants completed a background questionnaire consisting of sociodemographic questions including age, education and employment, previous cervical cancer screening experiences and potential barriers to screening (Table 1, Table 2 and Table 3). The 25-item questionnaire allowed assessment of changes in cervical cancer knowledge and attitudes after participating in the intervention, as well as movement toward uptake of cervical cancer screening. The presentation discussed cervical cancer causes, symptoms, risk and protective factors, and Jamaica Cancer Society’s screening recommendations. Culturally relevant images were incorporated to provide a dynamic tool for health promotion and to promote understanding of cervical cancer, and included the location of the cervix in a woman’s body, screening test procedures, and location of the nearest health facility for screening. At the end of the intervention, time was allowed for questions and a pamphlet with key cervical cancer information covered was distributed.

**Table 1:** Sociodemographic characteristics of intervention participants (*n* = 225).

Variables	*n* ^a^	% ^b^
*Parish*		
Hanover	51	22.7
St. James	80	36.4
Trelawny	31	13.8
Westmoreland	61	27.1
*Age (years)* ^**c**^		
18–29	93	41.33
30–39	31	13.78
40–49	36	16.0
50 and older	64	28.4
*Highest Education Level*		
No formal education	4	1.8
Primary (1–6 grade)	54	24.0
Secondary (8–11 grade)	117	52
College/Technical/Vocational	41	18.2
Graduate School	3	1.3
*Marital status*		
Single	139	61.8
Married	29	12.9
Living together	44	19.6
Divorced or separated	6	2.7
Widowed	4	1.8
*Occupation*		
Unemployed	123	54.7
Unskilled employment	47	20.9
Skilled laborer	13	5.8
Office work/professional	13	5.8
Business owner	17	7.6
Other	2	0.9
*Time to nearest clinic*		
30 min or less	149	66.2
Over 30 min	72	32.0
*Number of children* (mean, SD)	2.5 ± 2.4	

^**a**^ Totals may not equal 225 due to missing values; ^**b**^ Percentage totals exclude participants who omitted answering the question; ^**c**^ Mean age—37.8 years, SD±16.7, range 18–79.

**Table 2:** Previous cervical cancer screening experience.

Variables	*n* ^a^	% ^b^
*Participant’s CCa screening background*		
Previously heard about CCa screening	141	64.7
Previously screened for CCa	100	46.7
Knowledge of pap smear purpose	147	65.3
Participant has time for CCa screening	214	97.7
Aware of CCa screening location with free services	70	32.9
Aware of CCa treatment location	96	45.5
*Source of knowledge of CCa*		
Doctor/Nurse	133	62
Family members	35	16.3
Friends	36	16.8
Media	69	32.1
Other	14	6.5

^**a**^ Totals may not equal 225 due to missing values; ^**b**^ Percentage totals exclude participants who omitted answering the question; CCa: cervical cancer.

**Table 3:** Participant attitudes about cervical cancer screening.

Variables	Pre-Test		Post-Test		Difference	*p*-Value
	*n*	%	*n*	%	%	
Pap smear embarrassing	18	8.0	19	8.4	0.4	*p* = 0.9647
Pap smear painful	119	52.9	70	31.1	−21.8	*p* = 0.0903
*Reasons for never getting screened*	n	%	-	-	-	-
Afraid of pain	43	33.1	-	-	-	-
Afraid of bad results	20	15.4	-	-	-	-
Didn’t know about Pap smears	14	10.8	-	-	-	-
Too expensive	8	6.2	-	-	-	-
Don’t know where to get tested	6	4.6	-	-	-	-
Don’t see a need	22	16.9	-	-	-	-
Not sexually active	5	3.9	-	-	-	-
Clinic is too far	1	0.8	-	-	-	-
Other	47	36.4	-	-	-	-

### 2.2. Study Measures

Knowledge of cervical cancer screening tests was assessed via two items. Participants were asked, “can you name or describe a test used to check for cervical cancer?” Pap test and HPV DNA test were classified as correct answers. Participant knowledge of recommended frequency of cervical cancer screening was assessed by asking, “how often should women get screened for cervical cancer?” Participants could select “don’t know,” “more than once a year,” “once a year,” “once every 2 or more years,” or “only if they have symptoms,” with “once a year” classified as correct. Knowledge of causes of HPV infection was assessed by asking, “how can people become infected with what causes cervical cancer?” Participants could select, “yes,” “no” or “not sure” from a list of possible causes of infection (Table 4). Knowledge of cervical cancer symptoms and risk factors were assessed by asking participants to select “yes,” “no,” or “not sure” from a list of possible choices (Table 4).

**Table 4:** Knowledge of screening tests, risk factors, symptoms, prevention and intention to screen.

Variables	Pre-Test		Post-Test		Difference	*p*-Value
	*n*	%	*n*	%	%	
*Knowledge of CCa screening tests*						
Name of CCa screening test	58	30.2	155	80.7	50.5	*p* = 0.0312 *
Recommended frequency of screening	86	41.6	172	83.1	41.5	*p* < 0.0001 **
CCa is preventable	22	11	113	56.5	45.5	*p* < 0.0001 **
*Knowledge of cause of CCa infection* ^**a**^						
Skin to skin contact of genitals	72	35	192	93.2	58.2	*p* < 0.0001 **
Sexual intercourse	77	37.6	192	93.7	56.1	*p* < 0.0001 **
Kissing	149	74.1	151	75.1	1	*p* = 0.7893
Witchcraft	134	66	171	84.2	18.2	*p* < 0.0001 **
Consuming unsafe water/food	87	43.1	130	64.3	20.3	*p* = 0.2065
*Knowledge of CCa symptoms* ^**b**^						
Pelvic pain	89	43.6	188	92.2	48.6	*p* < 0.0001 **
Painful sex	84	40.6	189	91.3	50.7	*p* < 0.0001 **
Blood in vaginal discharge	75	36.4	191	92.7	56.3	*p* < 0.0001 **
Bleeding after menopause	102	50.3	191	94.1	43.8	*p* < 0.0001 **
Bleeding between menstrual cycles	97	47.3	185	90.2	42.9	*p* < 0.0001 **
Bleeding after sex	70	33.8	199	96.1	62.3	*p* < 0.0001 **
Burning sensation while urinating	45	22.2	51	25.1	2.9	*p* = 0.4142
*Knowledge of how to prevent CCa*						
Getting a pap smear	158	76.4	202	97.1	20.7	*p* < 0.0001 **
Using condoms	77	37	189	90.9	53.9	*p* < 0.0001 **
Being faithful to one partner	114	54.8	200	96.2	41.4	*p* < 0.0001 **
Delaying sex until after 16	105	51	197	95.6	44.6	*p* < 0.0001 **
Avoiding smoking	112	54.6	189	92.2	37.6	*p* < 0.0001 **
Getting the HPV vaccine	124	59.9	195	94.2	34.3	*p* < 0.0001 **
*Pre and post-test knowledge scores:*	Pre-test		Post-test			
*mean ± SD (range)*	10.6 ± 4.8 (0–20)		17.8 ± 2.5 (6–21)			
*Difference in post-test - pre-test knowledge index score*	*N*		Mean		95% CI	*t* = 21.8 (*p* < 0.0001 *)
	211		7.2		6.6, 7.9	

^**a**^ Participants correctly answered kissing, witchcraft, and consuming unsafe food/water do not cause HPV infection; ^**b**^ Participants correctly answered burning sensation while urinating is not a symptom of cervical cancer; * Denotes significance at 5% level; ** Denotes significance at 1% level; CCa: cervical cancer.

Intention to screen was assessed by asking participants to select “yes” or “no” to “have you ever thought about getting screened for cervical cancer?” and subsequently classifying them into one of the Stages of Change based on their answer. Participants who answered “no” were assigned to the pre-contemplation stage. Participants who answered “yes” were asked the follow-up question, “are you planning on getting screened for cervical cancer in the next 6 months?” “No” answers were assigned to the contemplation stage, and “yes” answers were assigned to the preparation stage.

The instruments were pilot-tested twice prior to data collection with a sample of 20 Jamaican women to ensure the presentation content and survey questions were clearly understood. After completion of the pre-test, interviewers administered the educational presentation, immediately followed by the post-test. The pre-test/post-test was self-administered, with interviewers assisting women who needed or requested assistance by reading each question and recording the participant’s answers. Intervention sessions were conducted one-to-one and in groups of up to 30 women, with an average of eight women per session. One-to-one sessions lasted approximately 30 min, with group sessions lasting up to 60 min. Presentations lasted approximately 15 min for both methods of delivery; due to staff limitations, women requiring assistance filling out the questionnaire during group sessions had to wait for staff to become available, thus the longer duration time for group sessions. Accordingly, each woman received the same amount of cervical cancer information, independently of delivery method. Participants were contacted approximately six months after completion of the intervention to determine the rate of cervical cancer screening uptake after participation in the study.

### 2.3. Ethics

The study protocol was approved by the Institutional Review Board of the University of Alabama at Birmingham, the Advisory Panel of Ethics and Medico-Legal Affairs in the Jamaican Ministry of Health, and the Western Regional Health Authority of Jamaica. All study participants gave written informed consent for the use of their de-identified data. Participants also provided consent to follow-up by phone at 6 months post-intervention, which we used to inquire if they had obtained a cervical cancer-screening test after completion of the intervention.

### 2.4. Statistical Analysis

Paired *t*-tests and tests for correlated proportions were conducted on pre-test and post-test scores to evaluate changes in cervical cancer knowledge and intention to screen post-intervention. A summated index of correct responses to knowledge of cervical cancer screening tests, causes of infection, symptoms, and risk factor questions was created. A paired t-test with α = 0.05 and 95% confidence intervals was used to assess differences between pre-test and post-test index scores. McNemar’s tests with α = 0.05 were used to assess the significance of differences between pre-test and post-test scores for knowledge variables included in the summated index and for the intention to screen variables. Data were analyzed using SAS software 9.3 [20].

## 3. Results and Discussion

### 3.1. Results

Table 1 lists participant demographic information. Sixty-five percent of participants had heard of cervical cancer screening and correctly identified the purpose of Pap tests prior to the intervention, however only one third knew the location of a health facility providing free Pap tests (Table 2). Most participants (66%) lived within 30 min from the nearest health center, with 28% of these living within 10 min of a health center.

The most commonly cited reason for never seeking screening was “Other” (36.4%)—with “never thought about it” as the predominant write-in answer—followed by “afraid it would be painful” (33.1%) and “don’t see a need” (16.9%) (Table 3). Ninety-seven percent of participants stated they would be able to take time out of their day to seek cervical cancer screening (Table 2). Less than half of participants (41.6%) were aware of the Jamaica Cancer Society’s recommendation of one Pap test per year at pre-test, increasing to 83.1% at post-test (*p* < 0.0001) (Table 4).

Although perception that a Pap test would be painful increased by 21.8 percentage points, and perception that a Pap test would be embarrassing decreased 0.4 percentage points, these differences were not significant (Table 3). Results show a significant increase in cervical cancer knowledge from pre-test to post-test (Table 4), with the mean number of correct responses increasing from 10.6 (SD ± 4.8, range 0–20) in the pre-test to 17.8 (SD ± 2.5, range 6–21) in the post-test. The number of women who responded that cervical cancer is preventable increased from 11% at pre-test to 56.5% at post-test (*p* < 0.0001). Approximately one third of participants in the pre-test correctly identified skin-to-skin genital contact and sexual intercourse as ways to acquire HPV infection, increasing to 93.2% and 93.7%, respectively, in the post-test (Table 4). Most knowledge items regarding cervical cancer symptoms increased significantly between pre-test and post-test (all reached significance at 1% level except “burning sensation while urinating”). Similarly, knowledge of items regarding cervical cancer prevention increased from 20.7 to 53.9 percentage points (*p* < 0.0001).

**Figure 1:**
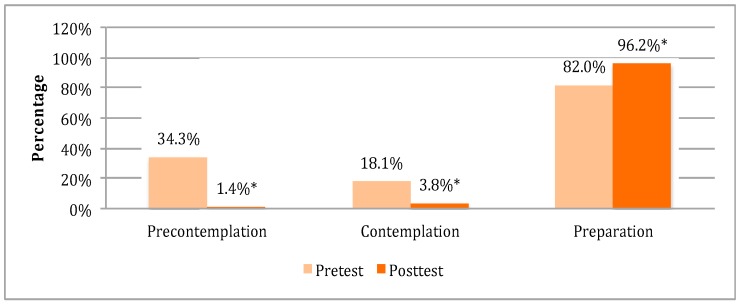
Distribution of respondents by Stages of Change at pre- and post-test (*n* = 210 for pre-contemplation; *n* = 133 for contemplation and preparation). * Denotes significance at 1% level.

We found an increase in intention to screen for cervical cancer from pre-test to post-test, with movement through the Stages of Change from pre-contemplation toward the action-oriented stage of preparation (Figure 1). The percentage of participants in the preparation stage, corresponding to participants stating intention to screen within the next six months, increased from 82% at pre-test to 96.2% at post-test (*p* < 0.0001).

Among the 123 participants we were able to contact for follow-up six months after the intervention, 50 (40.7%) were screened for cervical cancer, while 73 (59.3%) did not screen (Table 5). We used a subsample of participants reached post-intervention for which pre-intervention and post-intervention screening information was available (*n* = 97) to compare the post-intervention screening rate of women who had screened for cervical cancer 5 years or more prior to this intervention (*n* = 56), to that of women who had never screened prior to the intervention (*n* = 41). As expected if our intervention was successful in motivating screening-naïve women, we found no statistical significance between the two groups of women (two-tailed test *z* = 1.1, *p* = 0.29); if anything, screening-naïve women tended to have a higher screening rate 6 months post-intervention (19/41 = 46%) than women who had prior screening history (20/56 = 36%).

**Table 5:** Cervical cancer screening rates.

Variables	Participants Reached		Screened		Not Screened	
*Parish*	*n*	% ^**a**^	*n*	% ^**b**^	*n*	% ^**b**^
Hanover	19	37.3	9	47.4	10	52.6
St. James	54	67.5	19	35.2	35	64.8
Trelawny	15	48.4	9	60	6	40
Westmoreland	35	57.4	13	37.1	22	62.9
Cumulative	123	55.2	50	40.7	73	59.3

^**a**^ Percentage out of entire survey population; ^**b**^ Percentage out of women who were reached.

### 3.2. Discussion

Our findings suggest that a culturally sensitive theory-based educational intervention can be effective at improving cervical cancer knowledge and increasing intention to screen for cervical cancer among women in western Jamaica. There were statistically significant increases in the percentage of correct responses between the pre-test and post-test, and the majority of participants moved along the Stages of Change toward the preparation stage. Our results are comparable to other interventions employing similar methodologies that have resulted in significantly improved cancer awareness and enactment of positive health behavior change [21,22].

The intervention was effective at dispelling some misconceptions about the causes and symptoms of cervical cancer. The proportion of women who correctly answered that kissing, witchcraft (obeah), and consuming unsafe water or food are not causes of HPV infection increased from pre-test to post-test, although witchcraft was the only item exhibiting significant change.

Access to a health center does not appear to represent a major barrier to getting screened for cervical cancer among women in our study, as the majority of participants lived within 30 min of a health center providing free Pap smears, and women stated their ability to make time to attend a health center for screening. However, some women shared anecdotally their frustration with the public health system’s long delays in processing and communicating screening test results, stating they or someone they knew had not received results of their last test or had experienced long waits in receiving them. These experiences have contributed to a lack of trust in the public health care system.

Previous studies have found that women with a high level of cervical cancer prevention knowledge and awareness of screening facility location are more likely to seek screening [11]. Consistent with previous studies [8], most participants in our study had heard of cervical cancer screening prior to the intervention and knew the purpose of a Pap test, yet only about one third knew the location of a facility providing free Pap tests—thus although most women lived near a health center, most did not realize their nearest health center provided free screening. Only one third of participants knew the cause of HPV infection, and less than half knew the recommended frequency of screening. Despite possessing cursory knowledge of cervical cancer screening tests prior to the intervention, most women in our study lacked comprehensive knowledge of disease transmission, symptoms, risk factors, and screening location, information that may empower them to seek preventive care.

There is a need to address Jamaican women’s perceptions regarding cervical cancer and its prevention. At pre-test, most women in our study believed Pap tests were painful, and fear of pain was the second most cited reason for failing to get a Pap test. The number of women who believed Pap tests were embarrassing remained unchanged from pre-test to post-test. Despite significant increases from pre-test to post-test in the percentage of participants who believed cervical cancer was preventable, almost half of women (44%) at post-test still believed cervical cancer was not preventable. These results highlight the need to increase education efforts that promote the benefits of screening and address women’s anxieties about the procedure in ways that will motivate and keep them engaged in cervical cancer prevention. The negative experiences with the public health system, coupled with fear of testing and limited knowledge of the effectiveness of prevention efforts, may make women less likely to seek screening.

Although we made an effort to provide women with a wide list of choices for potential reasons why they have not sought screening, we found many women had difficulty fitting their experiences into those categories, and the most frequently selected answer for not screening was “other”. It is important to understand the context under which Jamaican women fail to seek cervical cancer screening. Future studies may consider adopting a qualitative approach, as the open-ended nature of qualitative research yields extensive narratives that may provide a more comprehensive picture of women’s experiences, helping inform the development of future interventions to promote screening.

Over one third of participants reached post-intervention had screened for cervical cancer. Almost half of women reached who never screened pre-intervention—and over a third of women reached with a screening history pre-intervention—moved to the action Stage of Change and screened for cervical cancer. These results suggest our intervention successfully motivated screening-naïve women to adopt the positive behavior change and reach levels akin to women who had screened previously. However, movement through the Stages of Change has been found to be cyclical [23], requiring several efforts before individuals may reach their goals, thus women will likely need further support to help them maintain the behavior change. 

Studies have shown healthcare provider recommendations may positively influence uptake of cervical cancer screening [3,16,24]. There is a need for a comprehensive strategy to educate women about cervical cancer and reduce barriers to facilitate engagement in screening and follow-up care after receiving abnormal test results. As doctors and nurses are already the principal source of cervical cancer screening knowledge for women in the study (Table 2), providers may play a pivotal role in encouraging women to adopt protective health behaviors, dispelling misconceptions of screening tests, and providing comprehensive health education to keep women engaged in their health.

This study had several limitations. Responses to questions such as intention-to-screen may be biased due to social desirability, and may have differed had screening tests been available immediately following the intervention. As with other cancer studies in Jamaica, we experienced loss to follow up post-intervention, as we were unable to reach many women via phone to assess screening rates, possibly due to local factors such as frequent changing of SIM cards and spotty network coverage. However, we were still able to reach the majority of participants. Issues with incomplete surveys reduced our sample size, however we do not believe this compromised the significance of our finding as we still obtained a sample representative of the population in the four western parishes sampled. Lastly, budget and time constraints did not allow for long-term follow-up of participants to assess long-term effects of the intervention on participant cervical cancer knowledge and screening behavior.

## 4. Conclusions

The use of a theory-based educational intervention significantly increased knowledge of cervical cancer risk factors, symptoms, and screening tests, and resulted in almost one half of participants contacted seeking screening post-intervention. Our results demonstrate the ability of an educational intervention to reduce barriers and elicit positive cervical cancer screening behavior, and may contribute to future efforts to increase cancer-screening rates in similar low-resource settings. Future research focusing on healthcare providers is needed to assess provider knowledge, attitudes, practices and training regarding cervical cancer screening. Alternative screening technologies such as visual inspection with acetic acid (VIA), which may reduce loss to follow up and the burden on government laboratories to process test samples given results are available immediately, should be considered by Ministry of Health officials. Health officials should also develop recommendations for enhancing healthcare worker capacity to promote cervical cancer education, screening and adherence to follow-up care.
